# Randomized multicenter follow-up trial on the effect of radiotherapy for plantar fasciitis (painful heels spur) depending on dose and fractionation – a study protocol

**DOI:** 10.1186/s13014-015-0327-6

**Published:** 2015-01-20

**Authors:** Henrik Holtmann, Marcus Niewald, Benjamin Prokein, Stefan Graeber, Christian Ruebe

**Affiliations:** Department of Radiotherapy and Radiooncology, Saarland University Hospital, Kirrberger Str. 1, D-66421 Homburg, Germany; Department of Oral and Maxillofacial Surgery, University Hospital of Duesseldorf, Moorenstr. 5, D-40225 Duesseldorf, Germany; Institute of Medical Biometrics, Epidemiology and Medical Informatics, Saarland University Hospital, Kirrberger Str. 1, D-66421 Homburg, Germany

**Keywords:** Painful heel spur, Plantar fasciitis, Low dose radiotherapy, Randomized multicenter trial

## Abstract

**Background:**

An actual clinical trial showed the effect of low dose radiotherapy in painful heel spur (plantar fasciitis) with single doses of 1.0 Gy and total doses of 6.0 Gy applied twice weekly. Furthermore, a lot of animal experimental and in vitro data reveals the effect of lower single doses of 0.5 Gy which may be superior in order to ease pain and reduce inflammation in patients with painful heel spur. Our goal is therefore to transfer this experimentally found effect into a randomized multicenter trial.

**Study design/methods:**

This was a controlled, prospective, two-arm phase III-multicenter trial. The standard arm consisted of single fractions of 1.0 Gy applied two times a week, for a total dose of 6.0 Gy (total therapy time: 3 weeks). The experimental arm consisted of single fractions of 0.5 Gy applied 3 times a week, for a total dose of 6.0 Gy (total therapy time: 4 weeks). Following a statistical power calculation, there were 120 patients for each investigation arm. The main inclusion criteria were: age > = 40 years, clinical and radiologically diagnosed painful heel spur (plantar fasciitis), and current symptoms for at least 6 months. The main exclusion criteria were: former local trauma, surgery or radiotherapy of the heel; pregnant or breastfeeding women; and a pre-existing severe psychiatric or psychosomatic disorder.

**Methods:**

After approving a written informed consent the patients are randomized by a statistician into one of the trial arms. After radiotherapy, the patients are seen after six weeks, after twelve weeks and then every twelve weeks up to 48 weeks. Additionally, they receive a questionnaire every six weeks after the follow-up examinations up to 48 weeks. The effect is measured using the visual analogue scale of pain (VAS), the calcaneodynia score according to Rowe and the SF-12 score. The primary endpoint is the pain relief three months after therapy. Patients of both therapy arms with an insufficient result are offered a second radiotherapy series applying the standard dose (equally in both arms).

This trial protocol has been approved by the expert panel of the DEGRO as well as by the Ethics committee of the Saarland Physicians’ chamber.

**Trial registration:**

Current trial registration at German Clinical Trials Register with the number DRKS00004458

## Background

### Principles

The painful heel spur was first described by Plettner et al. [1] summarizing radiological findings of exostoses situated at the plantar part of the calcaneus or at the insertion point of the plantar aponeurosis. Various authors give values for the incidence of 8 to 88% of an unselected population [2-4]. Risk factors described are old age, obesity, and foot or leg deformities. Histopathological findings are fibroostosis promoted by mechanical stress to the plantar aponeurosis, slowly and continuously growing into its insertion region [5]. In a more chronical stage of the disease, the degenerative changes cause a local inflammation of the plantar aponeurosis (plantar fasciitis), which should be well differentiated from – for example – rheumatoid arthritis.

### Clinical symptoms

Radiological diagnosed heel spurs often are completely asymptomatic. 16% of the patients with painful heel spurs have local pain getting worse over weeks to months under the heel, which can further extend to the foot or the lower limb. Local pressure to the medial edge of the calcaneus may be painful [5]. To our own experience, most of the symptomatic patients cannot stand or walk for a long time, the pain may be even worse during the first minutes of rest after a walk.

### Radiological findings

Conventional lateral x-rays of the calcaneus are gold standard in the diagnosis of a heel spur. They show a calcified spur at the inferior side of the calcaneus. The intensity of pain does not correlate to the radiological size of the spur. Additionally a local ultrasound examination can be performed in order to examine the swelling and irritation of the plantar fascia. A bone scan may be positive showing local inflammation which remits after successful therapy. A good diagnostic alternative to the scintigraphy seems to be the MR imaging because of its superior soft tissue contrast and good anatomical resolution. Typical in MR imaging is a thickening of the fascia and a surrounding soft tissue edema. In contrast-medium enhanced sequences intratendinous signal alterations of the fascia are often found.

### Conventional therapy methods

Ice, heat, ultrasound, radiofrequency, laser beams and extracorporal shock wave therapy have been applied, all of them with confined clinical success. Steroids and local anesthetics injected into the plantar fascia and oral analgetic medication (NSAID) are often prescribed. Immobilization of the foot using special splints and adjustable shoes were applied. Physiotherapy was performed [2,3]. Iontophoresis using dexamethasone was found to be superior to iontophoresis with placebo (NaCl) [6]. Extracorporal shock wave therapy yielded a complete pain relief in up to 68% of the patients [7]. A randomized trial published by Batt et al. [8] showed that better results were recorded by adding local immobilization of the foot in maximum dorsal flexion during night to a standard therapy (NSAID, splints) compared to this standard therapy alone. According to Powell et al. [9], a splint applied immediately after diagnosis was as effective as an application one month later. In further randomized trials mechanical therapy using ortheses was found better than application of local analgesics only, silicon shoe inserts were found superior to other shoe adjustments [10], functional foot ortheses were found to be more advantageous than simple heel splints [11]. Most of these methods and their results have been summarized in an actual Cochrane review [12].

### Surgery

It is general consensus that patients will only undergo surgery in case conservative therapy methods have not yielded sufficient pain relief. Heider et al. [13] report good to excellent results in 26 out of 28 feet after open surgery. Favourable results were recorded as well using endoscopic release of the plantar fascia [14]. Nevertheless, complications like fractures of the calcaneus [15] as well as negative biomechanical consequences of plantar fascia relief have been reported [16].

### Radiotherapy – experimental data

The anti-inflammatory effect of radiation therapy has been known for a long time and has been reported in numerous publications. Nevertheless, the exact ultra-structural mechanism is still unclear. Some of the more ancient models are discussed in [17-20].

In animal experiments, Steffen et al. noticed anti-inflammatory effects of low-dose radiotherapy (6Gy) on antigen-induced arthritis in rabbits [21]. Hildebrandt et al. have shown that low-dose radiation effects can be explained by an influence on molecular mechanisms and inflammation mediators [22-24]. New in-vitro investigations by Hildebrandt et al. 2003 and Rödel et al. 2002 [25,26] show a reduced iNOS protein expression and NO-production in macrophages. Latter investigation could also point that single doses of 0.3-0,5 Gy lead to a local maximum of apoptosis in macrophages and a reduced E-selectin-presentation on endothelium cells combined with an enhanced expression of TGFβ1 [26]. Hildebrand could also show in vitro that depending on modulation of cytokine-stimulated E-selectin-presentation leukocytes could less adhere to endothelium especially with single doses of 0.3-0.6 Gy. This finding also underlines the anti-inflammatory effect of low-dose therapy [27]. Additionally Gaipl et al. (2009) revealed a maximum of activity-induced cell death in polymorphnuclear cells by use of single doses of 0.3 Gy in radiotherapy [28]. Further anti-inflammatory effects of low-dose radiotherapy seem to be a reduced CCL-20-chemokine-expression, a reduced adhesion of granulocytes to endothelium cells and an enhanced activity of AP-1 DNA ligation strength [29,30]. An actual review article points out the induction of expression of the X-linked inhibitor of apoptosis and TGFβ1 and the reduced expression of E- and L-selectin, Interleukin-1 and CCL20 through macrophages and polymorphnuclear cells using single doses between 0.5 and 0.7 Gy [31]. Another new review article shows additionally high levels of hemioxigenase 1 (HO-1) and heat shock protein 70 (HSP 70) with maximum single doses of 0.5 Gy [32].

### Radiotherapy – clinical results

Numerous retrospective trials have shown that low-dose radiotherapy for painful heel spur has a good analgesic effect, pain relief has been noticed in 65–90% of the patients [17,20,33-36]. However, a certain placebo effect is still under discussion [33]. Goldie et al. examined this effect in 399 patients with painful conditions of the locomotor system. They found a response in 60% of the patients whether irradiated or not; these results made the effect of radiotherapy questionable [37]. The trial, however, has been criticized because of missing clearly defined endpoints: furthermore the therapy was started in an acute stage of the diseases and the authors did not wait for spontaneous pain remissions. In the meantime, several more modern trials have shown the analgetic effect of radiotherapy. Seegenschmiedt et al. [2] performed a randomized trial treating 141 patients (170 heels) for painful heel spur using orthovoltage, comparing three radiotherapy schedules: 1 Gy/fraction up to 12 Gy, 0.3 Gy/fraction up to 3 Gy and 0.5 Gy/fraction up to 5 Gy. The overall complete pain relief was reported in 67–72% of the patients. The best results were seen after a total dose of 5 Gy. These results were confirmed by Schäfer et al. using a telecobalt machine, they achieved a complete pain relief in 58% [33]. Heyd et al. used 6 MV photon beams of a linear accelerator, they noticed a frequency of pain relief of 69% [4]. The same author group published a prospective randomized trial recently [38] comparing the effect of a total dose of 3 Gy (single fraction 0.5 Gy twice weekly) to that of a total dose of 6 Gy (single fraction 1 Gy twice weekly). Radiotherapy was reported very efficient however a dependency from dose could not be noticed. Mücke et al. looked for prognostic factors for pain relief in a multicenter trial [39]. They found an overall response in 60.9%. Significant favourable prognostic factors for pain relief were a patient's age over 58 years, the use of megavoltage techniques and the number of therapy series required. Niewald et al. showed in actual prospective study that a total dose of 6 Gy with single doses of 1 Gy twice weekly is much more effective than a total dose of 0.6 Gy with single doses of 0.1 Gy concerning analgetic effect and showed that a total doses of 6 Gy makes this effect durable for at least 1 year [40]. After the start of this trial, Ott et al. [41] published their trial comparing the effect of six single doses of 1Gy (total dose 6Gy) to six single doses of 0.5Gy (total dose 3Gy). They found no difference between the arms.

### Radiotherapy – side effects and risks

Physicians of other specialities sometimes refuse to refer patients to radiotherapy because of the fear of local side effects such as impairment of gonad function or induction of malignancies. Neither local toxicity nor tumour induction have been reported yet [34,42,43]. The dose to the gonads is comparable to that after radiodiagnostic interventions [20,44-46].

### Radiotherapy – conclusion

Summarizing the data taken from the literature it can be concluded that low-dose radiotherapy for painful heel spur with total doses ranging from 3–12 Gy is effective in the vast majority of patients and the side effects are negligible. However, a placebo effect cannot be excluded totally. Thus, randomized trials (like the present one) using defined criteria and scores are necessary [4].

Nevertheless, outside Europe there is a general reluctance against radiotherapy for benign disease. One reason may be that trials comparing conservative approaches, radiotherapy and surgery are very rare.

Much more important to our current trial is, however, to translate the experimental findings that lower single doses may be more advantageous than higher ones into the clinic comparing two patient groups with the same total dose and different fractionation schedules (1.0 Gy vs. 0.5 Gy).

### General therapy remarks

At the moment a lot of competing therapy approaches for painful heel spur are processed. One can summarize them to conventional/conservative approaches, radiotherapy and surgery. Up to now a lack of definite evidence is still existing which method to choose. Additionally and despite all experimental and clinical findings concerning radiotherapy for painful heel spur a general reluctance against radiotherapy is exsisting unfortunately.

### Study design

#### Eligibility criteria

Inclusion criteria

- clinical evidence of painful plantar heel spur with an anamnesis during ≥ 6 month
- radiological proof of the spur (in conventional radiography)
- typical clinical symptomatic with tenderness on palpation at medial tuber calcanei
- typical functional deficit: impairment of painless walking distance
- Karnofsky performance index ≥ 70%
- age ≥ 40 years
- written informed consent

Exclusion criteria

- previous radiotherapy and/or trauma to the foot region (i.e. fracture, tendon rupture)
- rheumatic, arterial or venous diseases, manifest lymphatic edema of the concerned foot/leg
- pregnancy, breastfeeding (regulatory reasons)
- severe psychiatric disorder
- statutory maintenance in medical affairs by relatives

### Informed consent

Before enrolment into the study, an informed consent is to be obtained from all patients after detailed information and explanations concerning:

- the positive and negative aspects of the therapy (i.e. toxicity),
- alternative therapy methods,
- data protection issues,
- schedule of the study (follow-up examinations, time frame, etc.),
- autonomy of choice in participating into the study, all the time possibility to withdraw the consent without the need to communicate special reasons

### Initial diagnosis

- Obligate: standardized anamnesis, clinical-orthopedic examination, lateral x-ray imaging of metatarsus and heel with diagnosis of heel spur in the region of tuberculum mediale calcanei at the dorsal insertion of the plantar aponeurosis
- Facultative: Ultrasound illustrating inflammation of plantar aponeurosis, bone scan in case of negative x-ray, MR-tomography to demonstrate the process of plantar fasciitis or detect further symptoms (i.e. bone marrow edema).

### Therapy protocol

After recording anamnesis, clinical-orthopedic investigation, radiographic confirmation of the diagnosis, checking eligibility criteria and written informed consent, filling in the SF-12-, Calcaneodynia- and VAS score forms, patients are randomly assigned to either Arm A or B of this study.

- Arm A (standard dose): Total dose of 6Gy, single fractions of 1 Gy applied two times a week over three weeks (either Monday/Thursday or Tuesday/Friday)
- Arm B (experimental dose): Total dose of 6 Gy, single fractions of 0.5 Gy applied three times a week over 4 weeks (Monday/Wednesday/Friday)
- Consequently, the gap between the fractions within a week is one to two days.
- Follow-up examinations are performed every six weeks after radiotherapy filling in the Calcaneodynia-Score, SF-12 questionnaire and VAS-score. On weeks 6, 12, 24, 36 and 48 after radiotherapy, the patients get an additional physical examination, on weeks 18, 30, 42 they only receive the questionnaires.

In case of persisting or aggravating origin symptoms despite radiotherapy minimum twelve weeks after radiotherapy patients in both arms are offered a second treatment with the standard fractionated dose. The ideal time point will be chosen by the patient and the local radiooncologist together. Results of these patients are categorized as unsatisfactory and censored beyond the leaving date of the patient. Patients wishing the second therapy series later than 12 weeks after therapy will be taken into account in the final analysis.

After follow-up examination of all 240 patients (120 per arm) for 48 weeks the study will be closed and the final data analysis will be performed. Interim analyses will be performed after enrolment of 120 and 180 patients. In case of significantly different results in the groups or the publication of practice changing results from other authors which lead an ethical reappraisal of this trial the randomization of new patients will be stopped and the already randomized patients will get their follow-up examinations up to 48 weeks.

The use of analgesic drugs and physiotherapy is allowed during the trial. These will be recorded and taken in to account in the final analysis. Patients having been treated by shock wave therapy or surgery will be excluded from the analysis.

### Randomization and statistics

Randomization is performed as a block randomization by the statistician. Patients are assigned randomly to one of the therapy arms by equal possibility for randomization for both arms.

According to a previous trial published by Niewald et al. (40) and an actual statistical calculation before beginning this study 120 patients are required in each arm and have to be evaluated over 48 weeks (inclusive a drop-out rate of 10% of the patients) in order to detect a difference of 15% in VAS and Calcaneodynia-Score with a power of 80% (error probability of 5%).

Statistics (primary and secondary endpoints) are performed using the MEDLOG software package (Fa. Parox, Münster) after observing the patients for 12 and 48 weeks and are controlled by the statistician.

### Primary endpoints (measured at 12 and 48 weeks after therapy)

- SF-12 sum score [47,48]
- Calcaneodynia sum score [2,49]
- VAS self-assessment-score

The endpoints will be measured 12 and 48 weeks after radiotherapy, the results will be compared.

### Secondary endpoints

- SF-12 single score
- Calcaneodynia single score
- Event-free interval

### Radiotherapy methods

Radiotherapy is performed under orthovoltage conditions (200–250 kV) or high voltage conditions using a cobalt unit or high energy photons of linear accelerators (up to 6 MV without bolus, 10 MV with a bolus of 1 cm).

For orthovoltage therapy, a plantar direct field with tissue equivalent bolus material affixed around the heel is used (‘Essen technique’). The dose should be standardized to a fix reference point (i.e. in 5 mm tissue depth).

For cobalt units or linear accelerators isocentric lateral parallel opposing portals are used (isocenter: center of calcaneus).

The dose is computed either by individual calculation or by depth- dose curves/charts of the therapy machines. The target volume should involve the calcaneus, plantar aponeurosis and the pain point including a safety margin of 2 cm. Gonads have to be shielded as well as possible.

### Quality assurance

Methods to ensure quality are especially:

- Questionnaires are signed for accuracy by physician and physicist
- Visiting and personally initiation of all participating centers. Participating centers will additionally been asked to send x-rays, portal imaging pictures and therapy plans of randomly selected patients to the study center
- Data entered into database will be checked independently by two physicians

### Ethics

The trial protocol has been approved by the Ethics committee of the Saarland Physicians’ Chamber (number 14/07 on 09/05/2012). Furthermore, it has been approved by the board of directors of the DEGRO (German Society for Radiation Oncology).

### Accrual time

Accrual of patients has begun in February, 2013 and is planned to last three years.

### Present status of the trial

In the meantime 120 patients have been enrolled into study. The interim analysis is under discussion. Besides Saarland University Medical School, two further centers are actually enrolling patients.

## Abbreviations

AP-1: Activator protein-1
CCL: Chemocine ligand
cm: Centimeter(s)
DEGRO: German Society for Radiation Oncology
GCGBD: German Cooperative Group for Radiotherapy for Benign Diseases
Gy: Gray
iNOS: Inducible nitric oxide synthetase
kV: Kilovolt
MR: Magnetic resonance
MV: Megavolt
NO: Nitric oxide
pH: Pondus hydrogenii
SF-12: Short Form-12
TGFβ1: Transforming growth factor β1
VAS: Visual analogue scale
